# Prediction of Mechanical Properties of Void Defect-Containing *C_f_*/SiBCN Ceramic Matrix Composite Based on a Multiscale Analysis Approach

**DOI:** 10.3390/ma18092116

**Published:** 2025-05-05

**Authors:** Yuncan Pan, Xin Liu, Jianyao Yao

**Affiliations:** 1 College of Aerospace Engineering, Chongqing University, Chongqing 400044, China; panyuncan@cqu.edu.cn; 2 Beijing Institute of Astronautical Systems Engineering, China Academy of Launch Vehicle Technology, Beijing 100076, China; buaa_xin@126.com

**Keywords:** ceramic matrix composites, SiBCN, representative volume element, random defects

## Abstract

Carbon fiber-reinforced SiBCN ceramic matrix composite ($C_{f}$/SiBCN CMC) is emerging as a promising candidate for advanced thermal protection systems, owing to its superior thermal stability and notable ablation resistance. In this study, the mechanical properties of $C_{f}$/SiBCN CMC are investigated theoretically and experimentally. Utilizing a multiscale approach, a representative volume element (RVE) is developed to predict mechanical properties based on detailed microstructural characterization. The predictions derived from the RVE demonstrate agreement with the experimental findings. The experimental results show dispersion in the mechanical properties of $C_{f}$/SiBCN CMC. To investigate whether the dispersion of mechanical properties is associated with defects, this study examines the impact of the location, content, and size of defects on the mechanical properties of the $C_{f}$/SiBCN CMC. The analysis reveals that the location, content, and size of defect all impact the mechanical properties of the $C_{f}$/SiBCN CMC, with overall porosity having the most significant effect. When the porosity is constant, variations in defect location and size also contribute to the observed variability in the mechanical performance of the $C_{f}$/SiBCN CMC.

## 1. Introduction

The rapid advancement of the aerospace industry has imposed increasingly stringent demands on thermostructural materials [1,2,3]. Silicoboron carbonitride (SiBCN) ceramics exhibit excellent thermal stability, high specific strength, and exceptional high-temperature performance, demonstrating potential for application in aerospace and aviation industries [4,5,6,7]. Compared to ceramics such as Si_3_N_4_ and BN, SiBCN exhibits excellent high-temperature stability, oxidation resistance, and high-temperature creep resistance, and it maintains an amorphous structure even at elevated temperatures [8], making it an ideal material for high-temperature structural ceramics. Nevertheless, the practical applications of SiBCN ceramics are restricted by their inherent brittleness. By introducing carbon fibers, carbon fiber-reinforced SiBCN ceramic matrix composites ($C_{f}$/SiBCN CMC) can be prepared, resulting in materials that exhibit reduced brittleness and enhanced modulus compared with SiBCN ceramics [9,10,11]. Given their exceptional thermal stability and notable ablation resistance, $C_{f}$/SiBCN CMCs are anticipated to be viable candidates for high-performance thermal protection systems [12,13,14,15]. In recent years, thermal protection systems have faced elevated demands for load-bearing capacity in conjunction with thermal protection, thereby underscoring the need to investigate the mechanical properties of $C_{f}$/SiBCN CMC.

Studies on the mechanical properties of $C_{f}$/SiBCN CMC are relatively scarce, with the majority of research primarily concentrating on their preparation and the investigation of their flexural properties. Lee et al. [16] prepared $C_{f}$/SiBCN composites using polymer infiltration and pyrolysis (PIP) technique, which showed an average flexural strength of 255 MPa and no brittle fracture even at 2000 °C. Zhao et al. [17] prepared $C_{f}$/SiBCN CMC by PIP technique and obtained its flexural strength, flexural modulus, tensile modulus and tensile strength based on a three-point bending test and tensile test. Ding et al. [18] prepared a $C_{f}$/SiBCN CMC by a modified PIP process using liquid poly(methylvinyl)borosilazanes as a precursor, and the resulting $C_{f}$/SiBCN CMC had flexural strength and fracture toughness of 371 MPa and 12.9 MPa·m^1/2^, respectively. Jia et al. [19] investigated the fracture behavior and flexural strength of $C_{f}$/SiBCN CMC after oxidation at different times at high temperatures and found that $C_{f}$/SiBCN CMC showed good oxidation resistance and more stable flexural strength at 1200 °C and 1600 °C. Niu et al. [20] investigated the effects of sinter densification and fiber coating on the mechanical properties of CMCs and found that higher sintering temperatures significantly increased the flexural strength, Vickers hardness, and elastic modulus of the $C_{f}$/SiBCN CMC due to further sinter densification, which increased the matrix strength and fiber/matrix interfacial bond strength. Reports on the tensile properties, a critical mechanical characteristic of $C_{f}$/SiBCN CMC, are relatively limited, and studies addressing the shear properties of $C_{f}$/SiBCN CMC are even scarcer.

The preparation of CMC involves a multitude of intricate processes that lead to differences in the structure of the material, which contribute to variability in the mechanical properties of the resulting materials. Among these, defects introduced during the composite preparation process have been shown to significantly affect the mechanical properties of the composites [21,22]. The impact of defects on the mechanical properties of composites has been extensively analyzed in the existing literature. Ge et al. [23,24] proposed a modified Chamis model for calculating the elastic properties of fiber tow with pore defects and developed an RVE incorporating pore defects to predict the elastic constants of braided composites. The study also compared the results of two void-generating methods; the element model was compared based on the elements chosen and the void model was compared based on the explicitly constructed voids. However, the effect of defect size on the mechanical properties of the composites was neglected, and the defect sizes of the models created by the two methods were inconsistent. Sun et al. [25] comparatively analyzed the effect of different void sizes, locations and shapes on the coefficients of thermal expansion of the matrix by developing a void/matrix RVE and further investigated the effect of void ratio on the coefficients of thermal expansion of 3D $C_{f}$/SiC composites. This work considered the effect of void sizes, location and shapes on the coefficients of thermal expansion of the matrix, but due to the simplification of the modeling method, it was not possible to consider the effect of void sizes, location and shapes on the coefficients of thermal expansion of 3D $C_{f}$/SiC composites. Huang et al. [26] conducted a thorough investigation involving experimental studies, analytical models, and finite element models to accurately predict the mechanical properties of 3D composites with void defects. The RVE with void defects was established to investigate the effect of porosity on the stiffness of 3D composites. Similarly, the modeling of defects in this work is still based on repetitive random element selection and it has not been possible to investigate the effect of factors other than porosity on the stiffness of 3D composites.

Studies on the effect of defects on the mechanical properties of composites are mainly focused on the porosity. There is a relative scarcity of research addressing how the size and spatial distribution of defects affect the mechanical properties of composites. However, different material preparation processes can lead to different locations, content, and sizes of defects within CMC [27,28]. As can be seen from the pictures reported in the literature, the size of inter-tow defects in CMCs fabricated by the PIP process is small, while small defects also exist within the fiber tows. In contrast, the defects of CMCs fabricated by the technology of chemical vapor infiltration (CVI) process mainly appeared in the inter-tow and are of a larger size. Meanwhile, the mechanical properties of CMCs fabricated by different processes have different mechanical properties. To accelerate the development of CMC, it is necessary to study the influence of various factors of defects on the mechanical properties of CMC to guide the manufacturing process. In this paper, the modeling of defects with different locations, content and sizes is realized based on Python script (Python 3.7), and the degree of influence of these factors on the mechanical properties of composites is investigated. Materials’ properties play an essential role in the performance of the thermal protection system, and in order to meet its impact resistance, the material should have certain mechanical properties [29]. This work investigates the influence of various defect factors on the mechanical properties of CMC, which can help to set quality control thresholds for materials applied to thermal protection systems. For example, in order to ensure adequate impact resistance of thermal protection systems, the parameters of the defects of CMC should be within a certain range. The dominant factors of defects on the mechanical properties of CMC can also be targeted to improve the fabrication method, e.g., repeated infiltration can be used to reduce the porosity of the material [30], and sintering additives can be incorporated to reduce the size of the material defects [31].

The present work analyzes the impact of defects on the stiffness and strength of $C_{f}$/SiBCN CMC while ensuring the accuracy of the numerical simulation method employed. This paper is organized as follows: In Section 2, the numerical simulation of this work is described in detail and the numerical simulation results are compared with the experimental results. Section 3 discusses the effect of the location, content, and size of defects on the mechanical properties of $C_{f}$/SiBCN CMC. In Section 4, the predicted obtained mechanical properties of $C_{f}$/SiBCN CMC are used for failure analysis of typical thermal protected structure. In Section 5, some conclusions are drawn.

## 2. Methods and Validation

This section describes the details of the computational framework, including the modeling of the defect-containing fiber tow and the 5-harness satin CMC RVE, the boundary conditions, the loading method, and the setting of the progressive damage. Then, the numerical simulation results are compared with the experimental results.

### 2.1. Multiscale Modeling Considering Defects

The $C_{f}$/SiBCN CMC used in this study are provided by Shandong Industrial Ceramic Research & Design Institute and are fabricated by PIP process.

**Figure 1:**
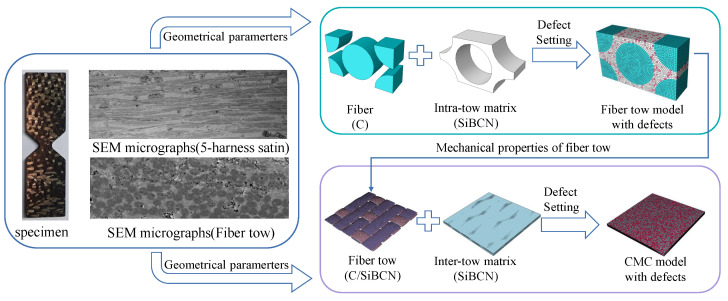
The multiscale modeling approach for 5-harness satin $C_{f}$/SiBCN CMC with defects.

**Figure 2:**
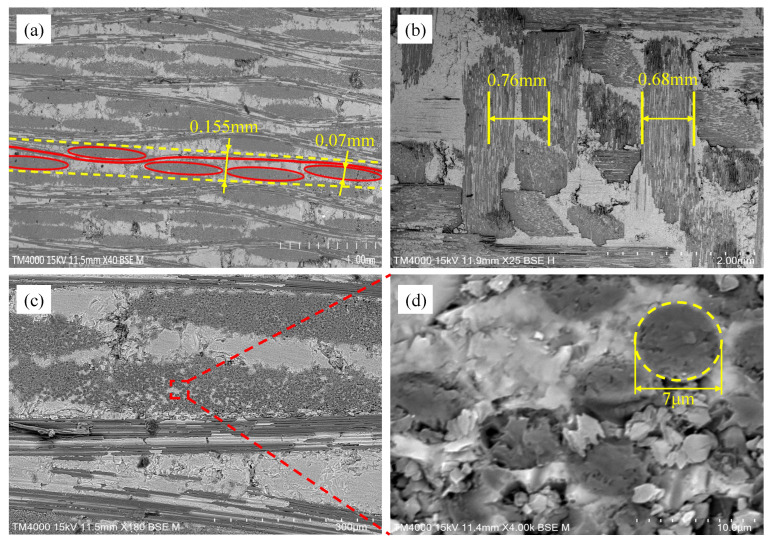
SEM micrographs of 5-harness satin weave $C_{f}$/SiBCN composite. (**a**) Specimen surface in in-plane direction; (**b**) Specimen surface in out-of-plane direction; (**c**) Enlarged image of a region in (**a**); (**d**) Enlarged image of (**c**).

Figure 1 illustrates the modeling concept of this work. Firstly, the RVE of the fiber tow and the 5-harness satin $C_{f}$/SiBCN CMC are established based on the geometrical parameters that have been obtained from the characterization. The micro-scale structures of several samples are characterized using scanning electron microscopy (SEM), as shown in Figure 2. Differences between individual yarns are neglected and the yarn cross sections are considered to be elliptical. The average values of each geometric feature are obtained after statistical analysis, as shown in Table 1.

The total porosity of the material was 23.82% and the fiber volume fraction of composite was 40%; these values were provided by the manufacturer. Based on the SEM image characterization, it can be determined that the ratio of intra-tow porosity to inter-tow porosity was about 1:7, so the intra-tow porosity was 2.98% and the inter-tow porosity was 20.84%. All other data in the Table 1 are the statistical averages values identified and obtained from the SEM images by the open-source software ImagicJ.

Then, the RVE was meshed and set up with defects. Next, the equivalent mechanical properties of the yarn (fiber tow) consisting of fibers and matrix were calculated based on the mechanics of the materials in Table 2. The strength properties of the materials in Table 2 are estimated values provided by the manufacturer. Finally, the equivalent mechanical properties of the 5-harness satin $C_{f}$/SiBCN CMC were predicted based on the calculated mechanical properties of the yarns and the matrix properties.

It should be noted that the defects were set up by setting the stiffness of the element to be very small. In this study, referring to previous studies [23,26,32], we set the stiffness of the element to 1 Pa. In this case, the element bore almost no load in the simulation, achieving an approximate simulation of a void.

**Table 2:** Mechanical properties of the constituent materials in composites [33,34,35].

Properties	C Fiber	SiBCN Matrix
$E_{11}$ (GPa)	230	117
$E_{33}$ (GPa)	15	117
$G_{12}$ (GPa)	9	48.75
$G_{23}$ (GPa)	5	48.75
$v_{12}$	0.25	0.2
$v_{23}$	0.3	0.2
$X_{T}$ (MPa)	2300	190
$X_{C}$ (MPa)	3000	1900
$Z_{T}$ (MPa)	1530	190
$Z_{C}$ (MPa)	1836	1900
$S_{xy}$ (MPa)	1000	50
$S_{yz}$ (MPa)	800	50

### 2.2. Defect Modeling Methodology

**Figure 3:**
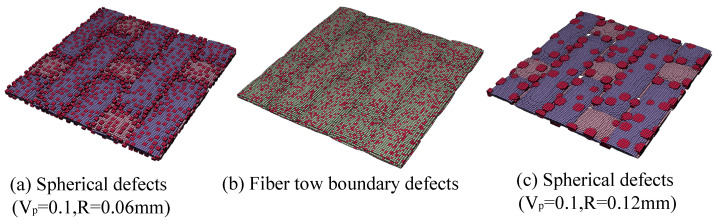
Finite element model with different types of defects.

The setting of the defects is based on a Python script developed in ABAQUS 2019. In prior defect modeling approaches, defects have been modeled by repeatedly selecting elements at random until the total volume of the selected elements reaches a set porosity. Therefore, prior defect modeling approaches can only control one factor of defect content (porosity). Compared to the previous work, the modeling method used in this work was able to set the location, content, and size of the defects, allowing them to be controlled by more factors and providing a more complete description of the defects in the CMC. The modeling of different types of defects is shown in Figure 3, where the elements in red indicate defects.

**Figure 4:**
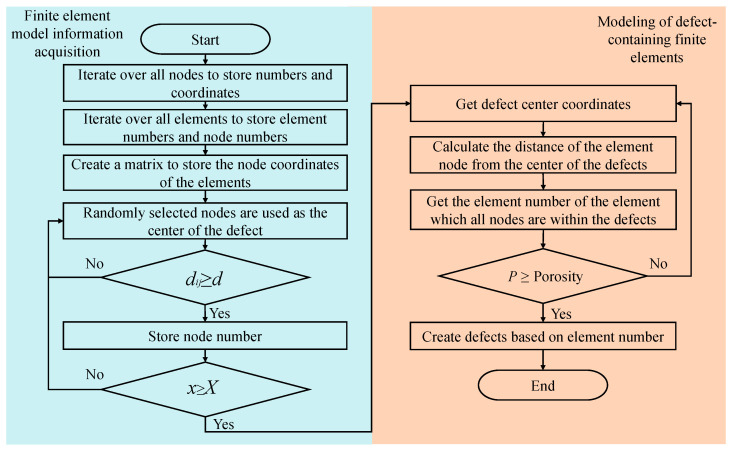
Modeling process for finite element model with defects.

The establishment of defects of different shapes can be realized by judging the distance between the nodes of the elements and the center of the selected defect. For example, the establishment of spherical defects requires judging whether the distance from all nodes of the element to the center of the defect is less than the radius of the spherical defect, and if it is, the corresponding element is selected to generate the spherical defect. Since the defects observed in the SEM of this work are mainly void defects, only spherical defects are generated in this work. In the subsequent analysis, only the effects of different factors on the mechanical properties of CMC are discussed when the defects are all spherical. The modeling flow is shown in Figure 4.

As shown in Figure 4, the modeling is divided into two parts in total: Acquisition of model information and defect modeling. All nodes of the RVE and their coordinates are stored first, and then all elements and their node numbers are stored. Then, a matrix is created to store the element numbers and their coordinates of the nodes. A node is randomly selected, and if its distance from all other selected nodes is greater than the set defect spacing d, the number of that node is stored. The step is repeated until the number of selected nodes exceeds the expected number of defects to be generated. Next, a determination of the distance between the nodes of the element and the center of the defect is performed. If the distances of all nodes of the element to a certain defect center are less than the size of the defect (meaning that the element is within the defect), the number of the element is stored. This step is repeated until the ratio of the total volume of all stored elements to the volume of the RVE can reach the porosity. Finally, these elements are set as defects.

In Figure 4, $d_{ij}$ is the distance between the currently selected random node *i* and the rest of the arbitrary nodes *j* that have been selected, and *d* is the distance between the center of the given defect and the center of the defect; *x* is the number of nodes that have been selected, and *X* is the expected number of the nodes that need to be selected; The *p*-value is defined as follows:

(1) $$P=\frac{\sum_{n=1}^{N}V_{n}}{V_{total}}$$

where *N* is the total number of selected elements, $V_{n}$ is the volume of the nth element, and $V_{total}$ is the total volume of the RVE.

For defects at the fiber tow boundary, it is sufficient to set the element selection area to the neighboring matrix elements of fiber tow.

### 2.3. Prediction of Equivalent Mechanical Properties

The equivalent mechanical properties of the composites are considered to be equal to the average mechanical properties of the periodic RVE. In the elastic state, the average stress and strain of the RVE are given by

(2) $$\bar{\varepsilon }=\frac{1}{V}\int_{V}\varepsilon dV$$

(3) $$\bar{\sigma }=\frac{1}{V}\int_{V}\sigma dV$$

where $\bar{\varepsilon }$ and $\bar{\sigma }$ are the average strain and stress of RVE, respectively, and *V* is the volume of the RVE.

It is assumed that the heterogeneous RVE is orthotropic and made from an elastic material. At this point, the equivalent intrinsic model can be written as follows:

(4) $$\bar{\sigma }=C\bar{\varepsilon }$$

(5) $$C=\left[\begin{array}{cccccc} c_{11} & c_{12} & c_{31} & \; & \\ c_{21} & c_{22} & c_{23} & \; & \\ c_{31} & c_{32} & c_{33} & \; & \\ \; & \; & & c_{44} & \\ \; & \; & & \; & c_{55}\\ \; & \; & & \; & & c_{66} \end{array}\right]$$

where C is the stiffness matrix of the RVE.

To obtain equivalent mechanical properties of the CMC, the RVE needs to be loaded under different working conditions to obtain the average stress and average strain of the RVE under loading. The periodic boundary condition ensures the continuity of the stress and displacement on the corresponding sides of the RVE, which can provide the exact distribution conditions of the stress distribution [36]. In the present work, the periodic boundary conditions proposed by Xia et al. [37] are introduced in the RVE. The general formula of periodic boundary conditions can be written as follows:

(6) $$u_{i}^{j+}-u_{i}^{j-}={\bar{\varepsilon }}_{ik}\Delta x_{k}^{j}$$

where the $u_{i}$ is the displacement field; ${\bar{\varepsilon }}_{ik}$ is the global average strain tensor; the superscript “j+” and “j−” denote the opposite and negative direction, respectively; and $\Delta x_{k}^{j}$ is a constant between each pair of surfaces, so the right side of Equation (6) is a constant, which can be achieved by imposing constraint equations on the nodes in finite element analysis. Similarly, constraint equations can be established for the corresponding edge nodes and corner nodes of the RVE. In the present work, as in the case of establishing random defects, the setting of periodic boundary conditions is realized based on Python scripts [38].

The elastic constants of the RVE can be obtained by applying six types of loading (uniaxial tension in *x*, *y*, and *z* directions, and shear in the xy, xz, and yz plane) to the RVE, respectively. For example, by applying uniaxial tension in *x*-direction, the mechanical properties in the *x*-direction can be calculated as follows:

(7) $$E_{x}=\frac{\bar{\sigma_{x}}}{\bar{\varepsilon_{x}}},\nu_{xy}=-\frac{\bar{\varepsilon_{y}}}{\bar{\varepsilon_{x}}},\nu_{xz}=-\frac{\bar{\varepsilon_{z}}}{\bar{\varepsilon_{x}}}$$

Similarly, other mechanical properties can be obtained.

### 2.4. Progressive Damage Model

In this paper, the Tsai–Wu criterion [39] is deemed appropriate to describe damage initiation of CMC, which is expressed as follows:

(8) $$F_{i}\sigma_{i}+F_{ij}\sigma_{i}\sigma_{j}=1\left(i,j=1,2,...,6\right)$$

where $F_{i}$ and $F_{ij}$ are coefficients associated with strength parameters and $\sigma_{i}$ represents the stress components. Expanding Equation (8), the formula can be written as follows:

(9) $$\begin{array}{cc} & F_{11}\sigma_{1}^{2}+F_{22}\sigma_{2}^{2}+F_{33}\sigma_{3}^{2}+2F_{12}\sigma_{1}\sigma_{2}+2F_{13}\sigma_{1}\sigma_{3}+2F_{23}\sigma_{2}\sigma_{3}\\ & +F_{44}\sigma_{4}^{2}+F_{55}\sigma_{5}^{2}+F_{66}\sigma_{6}^{2}+F_{1}\sigma_{1}+F_{2}\sigma_{2}+F_{3}\sigma_{3}=1 \end{array}$$

The specific equation for the coefficients associated with the strength parameters is as follows:

(10) $$\begin{array}{cc} \; & F_{11}=\frac{1}{X_{T}X_{C}},\;F_{22}=\frac{1}{Y_{T}Y_{C}},\;F_{33}=\frac{1}{Z_{T}Z_{C}}\\ \; & F_{44}=\frac{1}{S_{xy}^{2}},\;F_{55}=\frac{1}{S_{xz}^{2}},\;F_{66}=\frac{1}{S_{yz}^{2}}\\ \; & F_{12}=-\frac{1}{2\sqrt{X_{T}X_{C}Y_{T}Y_{C}}}\\ \; & F_{13}=-\frac{1}{2\sqrt{X_{T}X_{C}Z_{T}Z_{C}}}\\ \; & F_{23}=-\frac{1}{2\sqrt{Y_{T}Y_{C}Z_{T}Z_{C}}}\\ \; & F_{1}=\frac{1}{X_{T}}-\frac{1}{X_{C}},F_{2}=\frac{1}{Y_{T}}-\frac{1}{Y_{C}},\;F_{3}=\frac{1}{Z_{T}}-\frac{1}{Z_{C}} \end{array}$$

where X,Y,Z denote the strength in the x,y,z directions, and the subscripts T,C represent the tensile and compressive loading states, respectively; $S_{xy}$, $S_{xz}$,$S_{yz}$ denote the shear strength in the xy,xz,yz plane, respectively.

The exponential damage evolution model is used to describe the stiffness degradation of the CMC, using the damage variable *d* to indicate the degree of material degradation, which is expressed as follows:

(11) $$d=e^{-\frac{n}{c}}$$

where *n* denotes the number of times the material reaches the failure criterion (the material may still be able to meet the failure criterion again after the stiffness degradation in terms of stress); and *c* is used to control the extent of material stiffness degradation. In this work, the fiber stiffness degradation is 0.5 and the matrix stiffness degradation is 13.

When damage is initiated, the stiffness matrix of Equation (5) changes from C to C(d), which is given as follows:

(12) $$C\left(d\right)=\left[\begin{array}{cccccc} dc_{11} & dc_{12} & dc_{31} & \; & \\ dc_{21} & dc_{22} & dc_{23} & \; & \\ dc_{31} & dc_{32} & dc_{33} & \; & \\ \; & \; & & dc_{44} & \\ \; & \; & & \; & dc_{55}\\ \; & \; & & \; & & dc_{66} \end{array}\right]$$

Based on the user subroutine UMAT compiled in Fortran code, the above-described progressive damage model can be introduced into the simulation analysis of ABAQUS.

### 2.5. Validation

**Figure 5:**
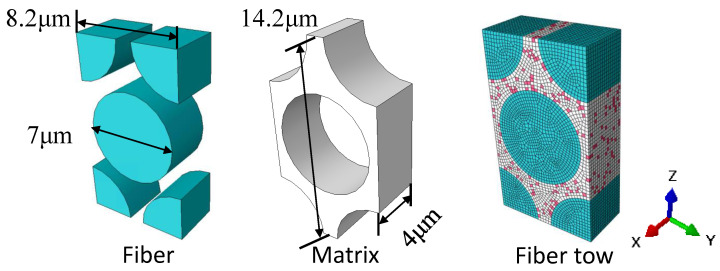
RVE of fiber tow.

**Figure 6:**
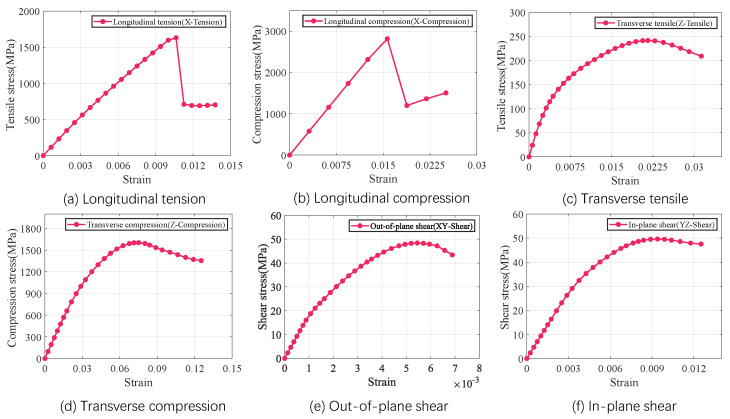
Stress−strain curves of the RVE of fiber tow under different load scenarios.

The RVE of fiber tow is developed based on the characterizing geometrical parameters in Table 1, as shown in Figure 5. The red elements indicate intra-tow void. The mechanical properties of the fiber tow material components are shown in Table 2. Boundary conditions are set and loads are applied to the RVE of the fiber tow, and the results of the finite element analysis are shown in Figure 6. The mechanical property parameters of the fiber tow mechanics extracted from the stress–strain curves of the fiber tow are summarized in Table 3.

**Figure 7:**
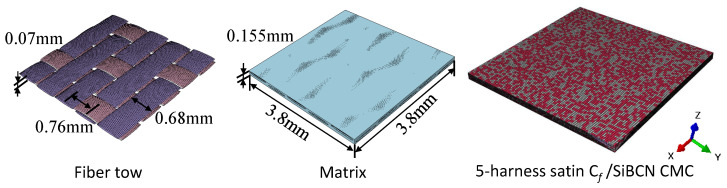
RVE of $C_{f}$/SiBCN CMC.

**Figure 8:**
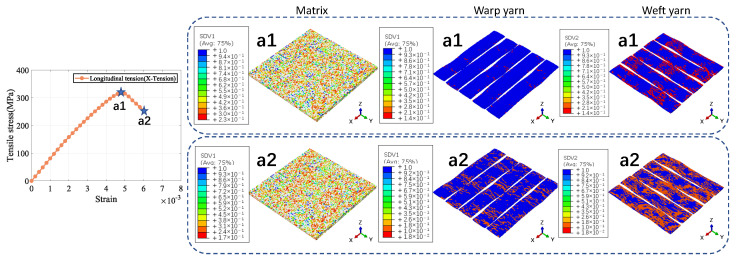
Longitudinal tension result of 5−harness satin $C_{f}$/SiBCN CMC.

**Figure 9:**
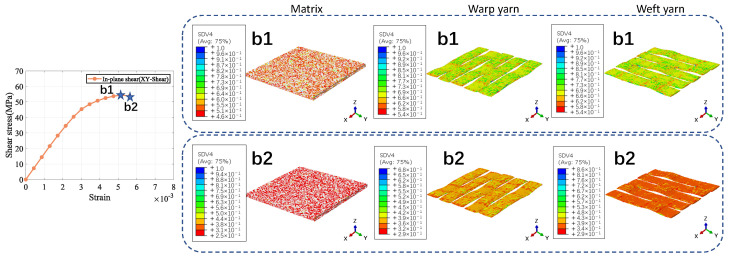
In-plane shear result of 5−harness satin $C_{f}$/SiBCN CMC.

As illustrated in Figure 7, the RVE of 5-harness satin $C_{f}$/SiBCN CMC is developed based on the characterizing geometrical parameters in Table 1. The red elements indicate inter-tow void. Axes 1, 2 and 3 of the $C_{f}$/SiBCN CMC correspond to the x, y and z axes in Figure 7. The RVE of the CMC is assigned the mechanical properties in Table 3 and subjected to finite element analysis, with the results of the stress–strain curves shown in Figure 8 and Figure 9. It should be noted that the above simulations have undergone mesh independence verification, and the results obtained have converged. Five models are generated and simulated with the same defect parameters, and the results do not differ by more than 0.3%, so only the results are shown for the line closest to the mean of the five.

The a1 and b1 star point in the figure represents the stage when the stress–strain curve reaches its highest point for the $C_{f}$/SiBCN CMC under loading. The blue part of the damage distribution map indicates no damage. The grayish-white part are all defects, and there is no damage in the defective part. The closer the color to red in the distribution map, the more severe the damage is, and the smaller the ratio of the mechanical properties to the mechanical properties before damage is.

Under longitudinal tensile loading, the matrix and weft yarn of the CMC are the first to produce damage due to the relatively small strength of the matrix and the transverse tensile strength of the fiber tow, as shown in Figure 8(a1). When the longitudinal tensile loading is so large that the damage to the warp yarns arises and expands, the stress–strain curve begins to decrease from the highest point, as shown in Figure 8(a2).

Similarly, the decrease in the stress-strain curve under shear loading is due to the damage of the $C_{f}$/SiBCN CMC as a whole, as shown in Figure 9(b1,b2). Under in-plane shear, the damage is more uniform for all components.

**Figure 10:**
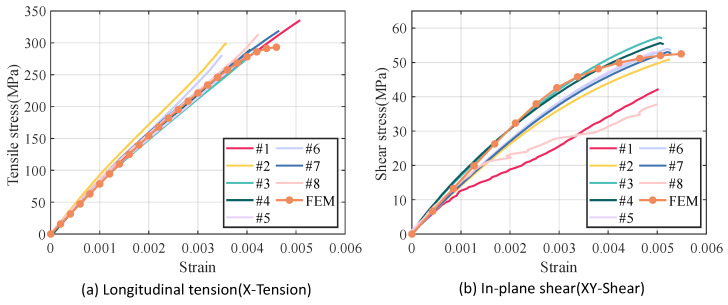
Comparison of finite element results with experimental results.

Figure 10a,b show the longitudinal tensile and in-plane shear finite element results of the 5-harness satin $C_{f}$/SiBCN CMC compared with the experimental results, respectively. As shown in Figure 10a, the finite element simulation results of longitudinal tension match well with the experimental results, and the trends of the stress–strain curves are consistent with the experiments. The finite element simulation results of in-plane shear show that the overall trend of the stress–strain curves obtained from the finite element analysis is consistent with the experimental results, as shown in Figure 10b. The predicted longitudinal tensile modulus and strength and in-plane shear modulus and strength obtained are within the range of experimental results.

The mechanical properties of $C_{f}$/SiBCN CMC obtained from the stress–strain graph in Figure 10 are shown in Table 4. Since the stress–strain curves of tension specimen 2, shear specimen 1 and shear specimen 8 are significantly different from those of the other specimens, their test data are ignored. The uncertainty of the experimental values is also listed in Table 4. From Table 4, it can be seen that the results of mechanical properties obtained from finite element analysis are in agreement with the experimental values The prediction of $E_{11}$ has the largest relative error from the mean of the experimental values, but this prediction is close to the lowest value of the experiment (78.0 GPa). The relative errors of the predictions of the other three mechanical properties from the mean of the experimental values are less than 3%.

## 3. Discussion of the Effect of Defects on Mechanical Properties

Different manufacturing processes lead to different location, content and size of defects in CMCs [27,28]. After calibrating the validity of the methodology in Section 2, this section investigates the effect of the location, content and size of defects on the mechanical properties of the 5-harness satin $C_{f}$/SiBCN CMC to explore the effect of defects on the dispersion of the mechanical properties of the 5-harness satin $C_{f}$/SiBCN CMC.

### 3.1. Effect of Intra-Tow/Inter-Tow Porosity Ratio on Mechanical Properties

From the experimental results in Figure 10 and Table 4, it can be seen that the mechanical properties of 5-harness satin $C_{f}$/SiBCN CMC are dispersive. To explore whether the defects are linked to the above experimental phenomena, the present work first investigates whether the intra-tow/inter-tow porosity ratio affects the mechanical properties of $C_{f}$/SiBCN CMC.

With the constant size of the RVE and a total porosity of 23.82% for the $C_{f}$/SiBCN CMC, the percentage of defects in intra-tow and inter-tow is redistributed. The model parameters for different intra-tow/inter-tow porosity ratios are shown in Table 5. Because it is difficult to have all defects within fiber tow, this work sets the porosity of intra-tow to be the same as the porosity of inter-tow for the case of the most intra-tow defects, and the defects are only present in the inter-tow for the case of the least intra-tow defects.

**Figure 11:**
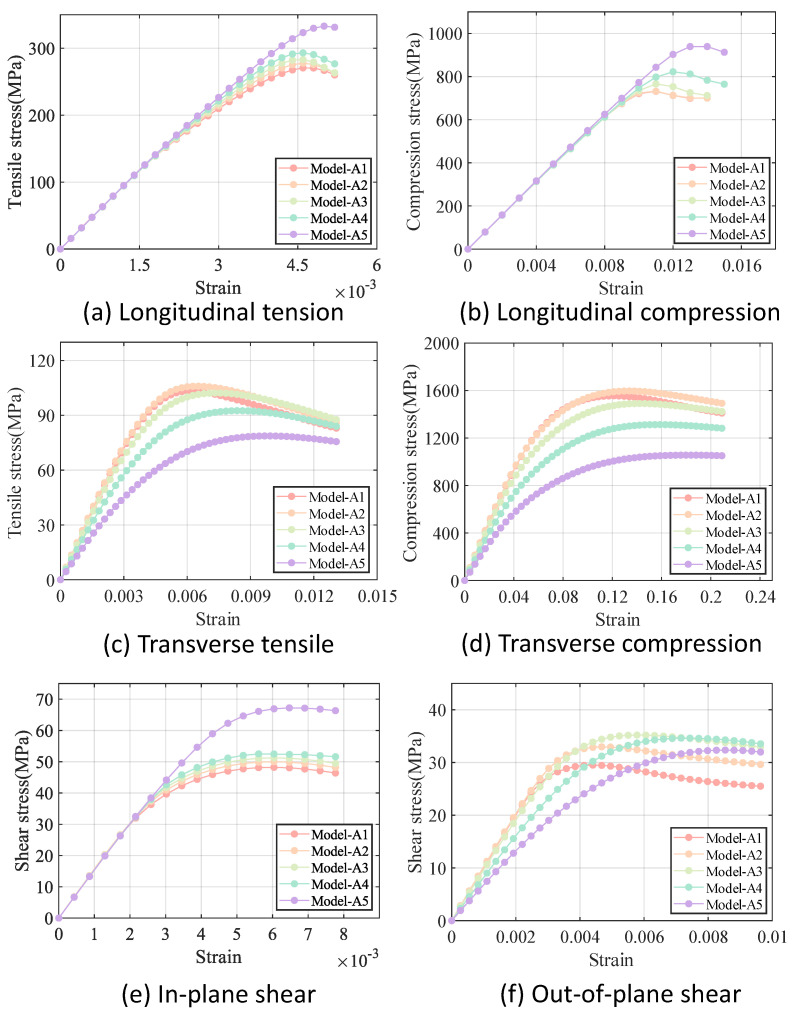
Stress−strain curves for models with different intra-tow/inter-tow porosity ratios.

It should be noted that five models are generated for each of the intra-tow/inter-tow porosity ratios. Since the stress–strain curves of the five models with the same intra-tow/inter-tow porosity ratios nearly overlapped, with little dispersion, only one curve is retained for the results of each of the intra-tow/inter-row porosity ratio. The comparison of the results of different models under different loading methods is shown in Figure 11. The mechanical properties of each model are shown in Table 6.

As can be seen in Figure 11, the difference in stiffness between the five models is smaller under longitudinal loading and in-plane shear loading compared to the difference in stiffness under transverse loading and out-of-plane shear loading. The longitudinal modulus and in-plane shear modulus of $C_{f}$/SiBCN CMC are less sensitive to changes in the intra-tow/inter-tow porosity ratio.

In the case of constant total porosity, there is a general trend that the greater the inter-tow porosity, the smaller all of the stiffnesses of the $C_{f}$/SiBCN CMC except $v_{23}$. The longitudinal and in-plane shear strengths of $C_{f}$/SiBCN CMC are directly proportional to the inter-tow porosity and the transverse strength is inversely proportional to the inter-tow porosity. The out-of-plane shear strengths of $C_{f}$/SiBCN CMC do not appear to be significantly related to changes in the inter-tow porosity.

### 3.2. Effect of Porosity on Mechanical Properties

From the analytical results in Section 3.1, it can be found that the intra-tow/inter-tow porosity ratio leads to changes in the mechanical properties of $C_{f}$/SiBCN CMC, provided that the total porosity remains constant. The inconsistency in the magnitude of the effect of porosity on the mechanical properties of fiber tow and $C_{f}$/SiBCN CMC may be the reason for this result.

To study the effect of porosity on the mechanical properties of fiber tows and $C_{f}$/SiBCN CMCs, RVEs of fiber tows and $C_{f}$/SiBCN CMCs with porosity from 0 to 20% are established in this paper. Among them, three RVEs are established for each level of porosity to investigate whether there is dispersion in the mechanical properties under the same porosity.

**Figure 12:**
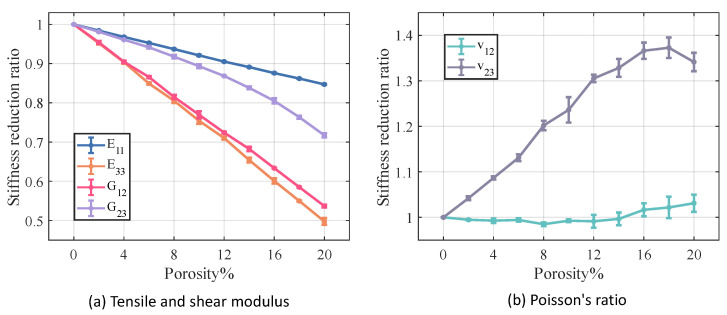
Effect of porosity on the stiffness of fiber tow.

**Figure 13:**
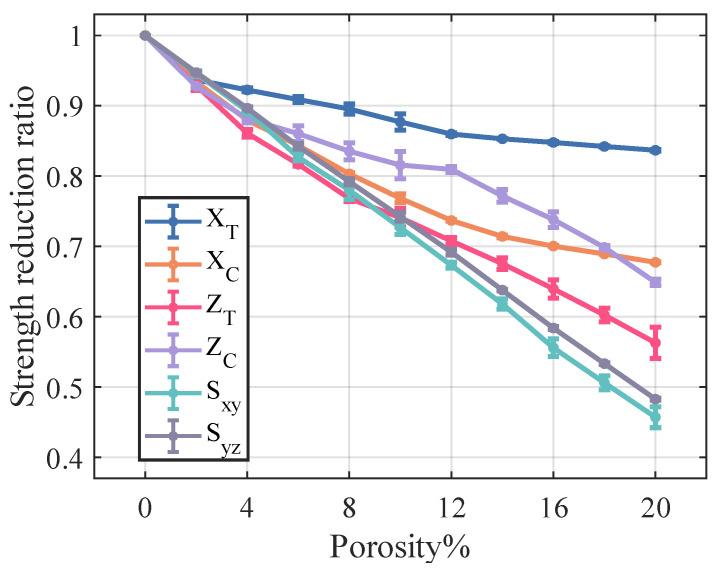
Effect of porosity on the strength of fiber tow.

Figure 12 and Figure 13 illustrate the variation in stiffness and strength reduction ratio of the fiber tow as a function of porosity. The mechanical properties of fiber tow, except Poisson’s ratio, decrease with increasing porosity.

Among the elastic properties, the $E_{33}$ and $G_{12}$ of the fiber tow are the most sensitive to changes in porosity, and when the porosity came to 20%, both the $E_{33}$ and $G_{12}$ of the fiber tow decrease to nearly half of their original values. In the case of equal porosity, the variability in the modulus of the fiber tow is relatively low, whereas the dispersion in the relative Poisson’s ratio is noticeably higher.

The $Z_{T}$,$S_{xy}$,$S_{yz}$ of fiber tow are more sensitive to changes in porosity, which may be related to the fact that these strength parameters are dominated by the matrix strength. Moreover, at a constant porosity level, the dispersion of the strength of the fiber tow is slightly greater than that observed in the stiffness.

**Figure 14:**
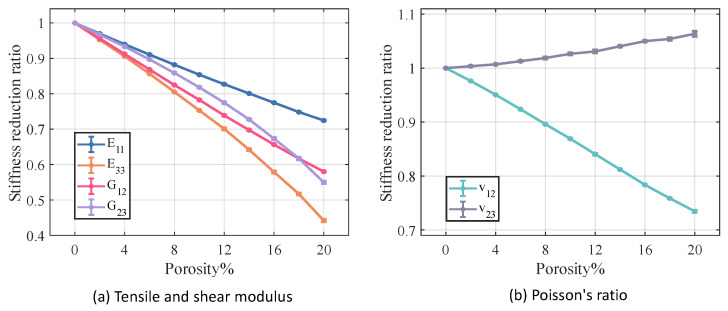
Effect of porosity on the stiffness of $C_{f}$/SiBCN CMC.

**Figure 15:**
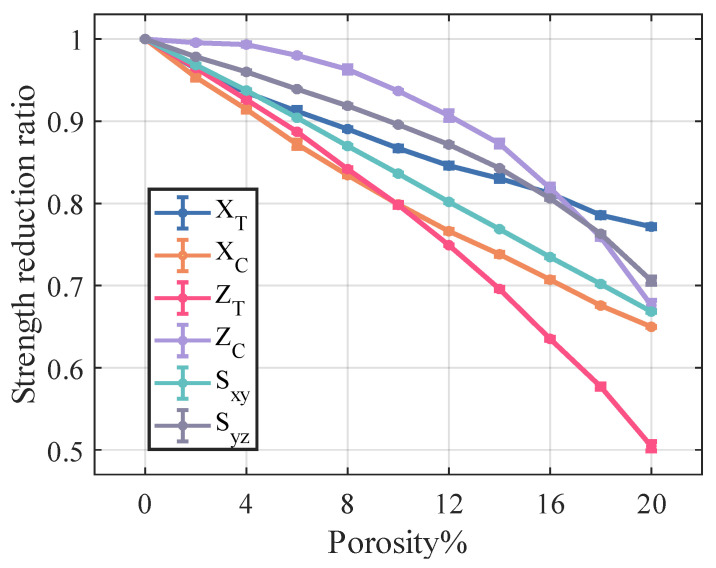
Effect of porosity on the strength of $C_{f}$/SiBCN CMC.

Figure 14 and Figure 15 illustrate the relationship between porosity and the mechanical properties of $C_{f}$/SiBCN CMC. Except for $v_{23}$, which is proportional to the increase in porosity, the rest of the mechanical properties of $C_{f}$/SiBCN CMC are inversely proportional to the increase in porosity. Similarly, when the porosity is constant, only $v_{23}$ has significant dispersion at high porosity, and the rest of the mechanical properties of $C_{f}$/SiBCN CMC are stable with minimal dispersion.

As the porosity increases, the overall decreasing trend of $C_{f}$/SiBCN CMCs is larger than that of fiber tows except for the $G_{12}$ modulus, which may be one of the reasons why the defects are smaller in the modulus with a larger percentage of $C_{f}$/SiBCN CMCs when the total porosity is constant in the previous subsection.

Compared with fiber tow, the strength of $C_{f}$/SiBCN CMCs show a more obvious trend of linear decrease or rapidly nonlinear decrease, which may be due to the smaller dispersion of the strength of $C_{f}$/SiBCN CMCs at the same porosity.

The $E_{33}$ and $Z_{T}$ of $C_{f}$/SiBCN CMC are most affected by porosity in stiffness and strength, respectively. Compared to fiber tows, $v_{12}$ of $C_{f}$/SiBCN CMC is much more sensitive to porosity changes.

### 3.3. Effect of the Percentage of Defects at the Fiber Tow Boundary on Mechanical Properties

Even if the porosity is the same, the mechanical properties of $C_{f}$/SiBCN CMCs are not identical, as demonstrated by the results in Section 3.2. This phenomenon of dispersion in the mechanical properties is more evident in the strength than in the stiffness, so it is possible that the different regions of stress concentration due to the different defects make the materials’ strengths differ.

**Figure 16:**
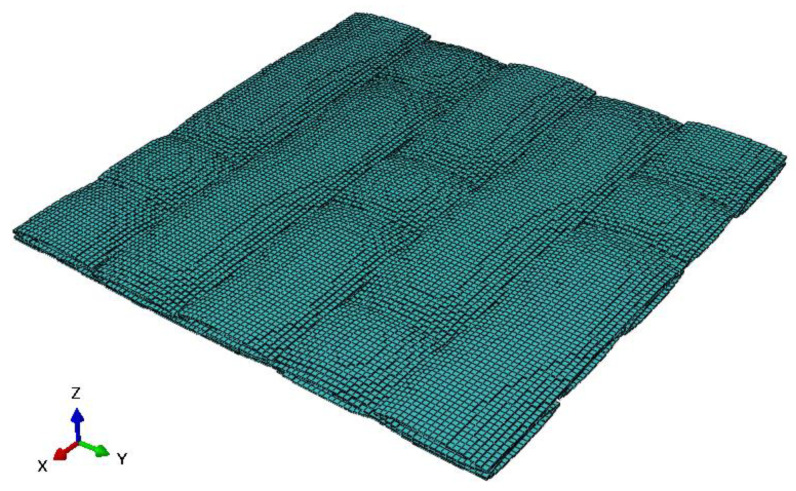
Selection of fiber tow boundary elements.

The presence of defects near the interface between the matrix and the fiber tows may make the effect of stress concentration in the material greater. To investigate whether the percentage of defects near the interface affects the mechanical properties of fiber tows and $C_{f}$/SiBCN CMCs, five RVEs for $C_{f}$/SiBCN CMCs are developed in this work, which all have a total porosity of 10%, with the difference being that the defects are distributed in a different proportion of the location, as shown in Table 7. The neighboring matrix elements of fiber tows (yarns) are selected as boundary elements, as shown in Figure 16.

**Figure 17:**
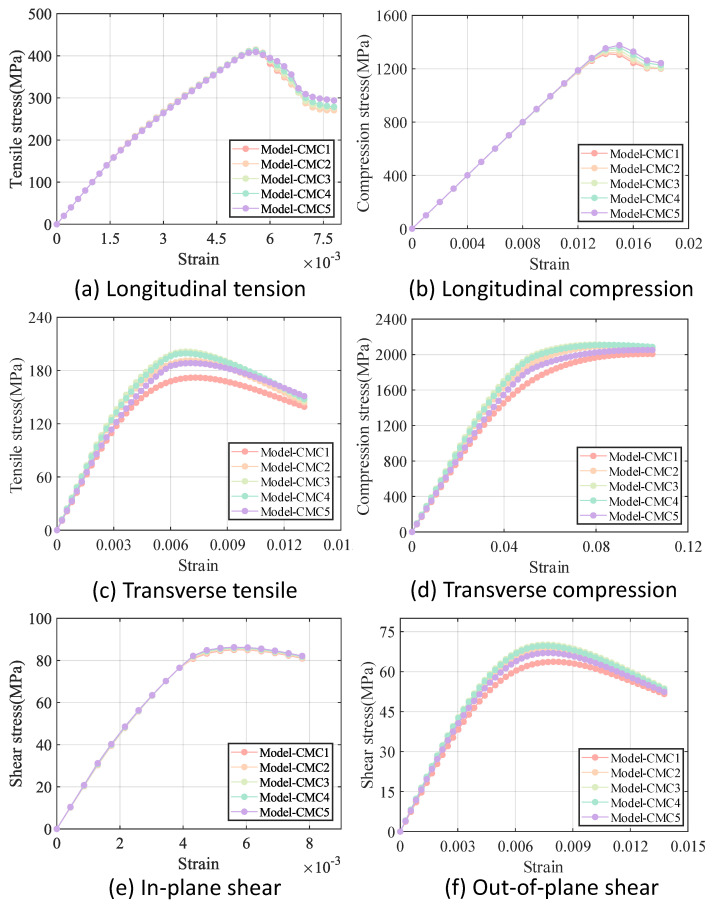
Stress−strain curves for $C_{f}$/SiBCN CMC with different porosity of boundary.

Five models are similarly generated for each fiber tow boundary porosity. Since the difference between the results of the five models with the same porosity of fiber tow boundary remains small, only one result is retained for each model. Figure 17 displays the results for various $C_{f}$/SiBCN CMC models subjected to different loading methods, while Table 8 provides a summary of the mechanical properties of each model.

From Figure 17 and Table 8, it can be seen that the $G_{12}$, $X_{C}$, and $S_{xy}$ of $C_{f}$/SiBCN CMC are positively proportional to the boundary porosity. The $E_{11}$ of the $C_{f}$/SiBCN CMC is related to the homogeneity in the distribution of defects is inversely proportional, and $X_{T}$ of $C_{f}$/SiBCN CMC are inversely proportional to the former and are greater at boundary porosities near 5%.

The $E_{33}$, $Z_{T}$, $Z_{C}$ and $S_{yz}$ of $C_{f}$/SiBCN CMC are all maximum at 5% boundary porosity except $G_{23}$ of $C_{f}$/SiBCN CMC which has been increasing with the increase in boundary porosity. It is possible that the more uniform distribution of defects prevents the defects at the boundary from accumulating to form large defects, which in turn improves the mechanical properties.

The Poisson’s ratio of $C_{f}$/SiBCN CMC is relatively stable; all components decrease and then increase with the increase of the percentage of boundary defects. The dispersion of Poisson’s ratio of $C_{f}$/SiBCN CMC is smaller, and the dispersion of $V_{23}$ is slightly larger than that of $V_{12}$.

### 3.4. Effect of Defect Size on Mechanical Properties

In Section 3.3, there is a phenomenon in which the mechanical properties of the material are smaller when the defects are fully distributed on the boundary. The more concentrated distribution of defects means that large defects are more likely to occur, and the mechanical properties of the material are related to the distribution of defects and probably also to the size of the defects.

To examine the impact of defect size on the mechanical properties of and $C_{f}$/SiBCN CMC, this study develops RVE of $C_{f}$/SiBCN CMC with defect sizes ranging from 0.09 mm to 0.21 mm, incremented at 0.03 mm intervals. Three models of each size are built to account for the dispersion of results.

**Figure 18:**
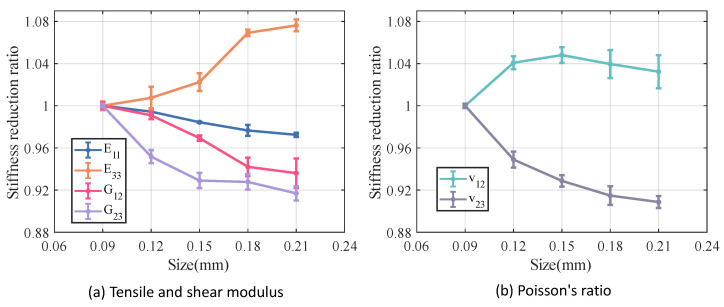
Effect of size of defect on the stiffness of $C_{f}$/SiBCN CMC.

**Figure 19:**
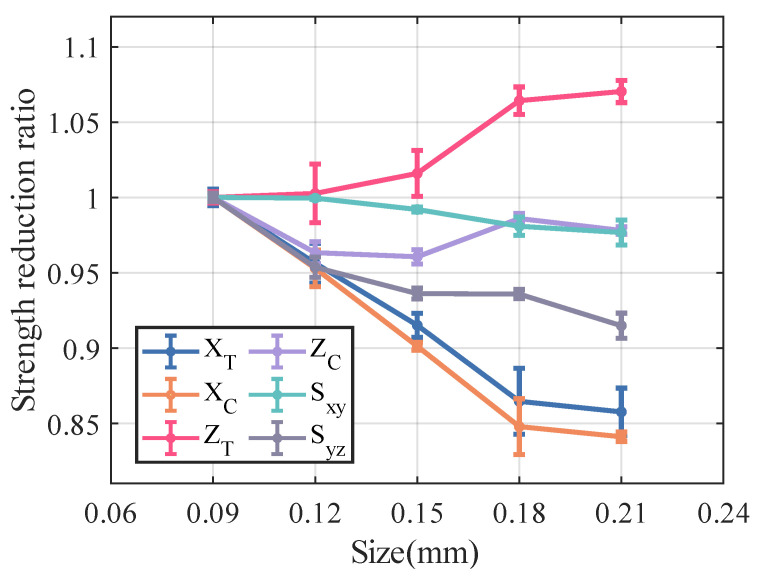
Effect of size of defect on the strength of $C_{f}$/SiBCN CMC.

Figure 18 and Figure 19 illustrate the variation in the mechanical properties of $C_{f}$/SiBCN CMC as a function of increasing defect size. The analyzed results indicate that most of the mechanical properties of $C_{f}$/SiBCN CMC decrease with increasing defect size. However, the $E_{33}$, $Z_{T}$ of $C_{f}$/SiBCN CMC increases with the increase in defect size, and there is no obvious pattern in the variation of $Z_{C}$, and this phenomenon may be due to the boundary artifacts. This phenomenon may be related to the modeling of CMC, which has thin layers. Since the radius of the defects is close to or larger than the thickness, the increase of the defect size is mainly presented in the longitudinal direction rather than in the transverse direction (thickness direction). This leads to the variation of the transverse mechanical properties of CMC with increasing defect size is different from the other mechanical properties.

Overall, excessive defect size leads to a deterioration in the elastic properties of the $C_{f}$/SiBCN CMC. Among these, the $G_{23}$ of the $C_{f}$/SiBCN CMC is significantly affected by increasing the size of the defect, while the $E_{11}$ shows minimal sensitivity to defect size.

Compared to the longitudinal modulus $E_{11}$, the tensile strength $X_{T}$ and compressive strength $X_{C}$ of the $C_{f}$/SiBCN CMC exhibit more pronounced variations as the size of defect increases, showing a decreasing trend. The $S_{xy}$ of the $C_{f}$/SiBCN CMC appears to be insensitive to changes in defect size. Although it exhibits some scatter, the overall variation is minimal.

## 4. Failure Analysis of Thermal Structure

**Figure 20:**
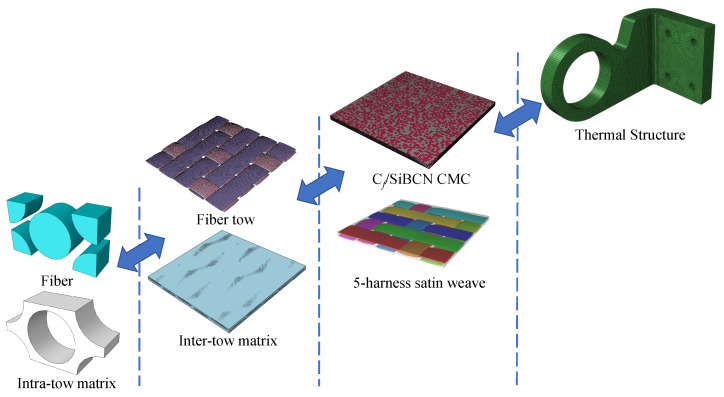
Application of multiscale analysis approach in real engineering case.

In this section, we describe the application of multiscale analysis approach to typical thermal structure. In recent years, practical engineering requires thermal structures to have adequate load-bearing capacity along with thermal protection. To perform a failure analysis of a thermally protected structure, it is necessary to obtain the mechanical properties of the CMC used in the structure. However, it is difficult to obtain all the mechanical properties of CMC in an experimental setting. In this case, it is necessary to obtain the mechanical properties of the CMC through a multiscale analysis method. As shown in Figure 20, based on the mechanical properties of the fiber and matrix and the microgeometric parameters of the CMC, the mechanical properties of the CMC are finally obtained for the failure analysis of the thermal structure.

**Figure 21:**
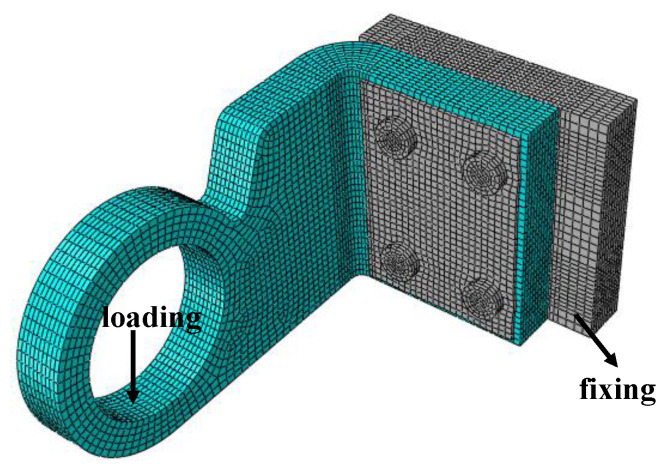
Schematic diagram of thermal structure.

A schematic diagram of a typical thermal structure is shown in Figure 21, including the loads applied to it and the boundary conditions. Among them, the gray part is the metal structure, which is used to fix the thermal structure. The damage of the metal structure is not considered in the analysis, only the progressive damage of the thermal structure (made of $C_{f}$/SiBCN CMC) is considered.

**Figure 22:**
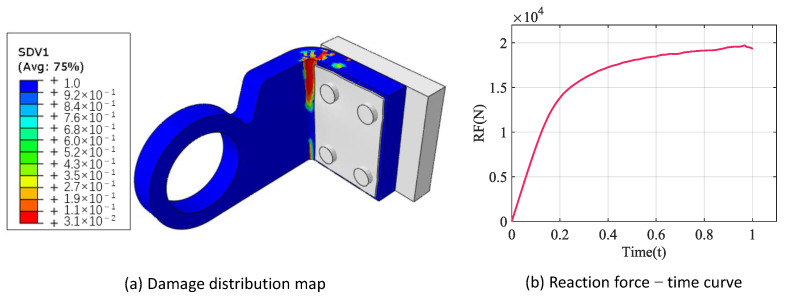
Failure analysis result of thermal structure.

In Section 2.5, we have verified the validity of the multiscale analysis approach by predicting the obtained tensile and shear properties in agreement with the experimental values. All the mechanical properties of the CMC are shown in Table 9. The mechanical properties in Table 9 are assigned to the thermal structure and the loads and boundary conditions are set up for failure analysis. The results of the failure analysis are shown in Figure 22.

Figure 22a shows the damage distribution cloud, the blue part indicates no damage, and the closer the color is to red, the more serious the damage is. The ultimate load of the thermal structure, i.e., the peak value of the reaction force–time curve is 19.7 kN, as shown in Figure 22b. It should be clarified that the experiment only provides the damage location of the structure with the maximum load that the structure can withstand. The damage location shown in Figure 22a is consistent with the experiment, which provides the maximum load applied to the structure as 20.4 kN.

As shown in Table 10, the results of the failure analysis of the thermal structure are close to the experimental value. Meanwhile, the predicted result is more conservative relative to the experimental value, which is in line with engineering applications. On the basis of the above, it is proved that the multiscale analysis approach is reliable in practical engineering application.

## 5. Conclusions

The RVEs for $C_{f}$/SiBCN CMC are developed utilizing a multiscale analysis approach to predict their mechanical properties. The finite element analysis results obtained from the RVE exhibited concordance with the experimental findings. The mechanical properties of $C_{f}$/SiBCN CMC are used in the failure analysis of a typical thermal structure, and the obtained ultimate load and failure form of the thermal structure are consistent with the experiment. Furthermore, the study investigated the impact of location, content, and size of defects on the mechanical properties of $C_{f}$/SiBCN CMC. Based on the finite element analysis results, the following conclusions are drawn:

(1) With the same total porosity, the greater the porosity of inter-tow, the smaller all modulus of $C_{f}$/SiBCN CMC are, with the shear modulus $G_{12}$ and $G_{23}$ having a greater reduction than the tensile modulus $E_{11}$ and $E_{33}$. The longitudinal tensile strength $X_{T}$, longitudinal tensile strength $X_{C}$, shear strength $S_{xy}$, and shear strength $S_{yz}$ of $C_{f}$/SiBCN CMC are, in general, positively correlated with the increase of porosity between clusters, whereas the transversal tensile strength $Z_{T}$ and the transversal tensile strength $Z_{C}$ are opposite.
(2) The increase in porosity leads to a significant loss of mechanical properties of fiber tows and $C_{f}$/SiBCN CMC. Among them, $E_{33}$,$G_{12}$,$S_{xy}$ and $S_{yz}$ of fiber tow are more sensitive to the change of porosity, and only about half of the initial properties of these properties remain when the porosity of fiber tow reaches 20%. Similarly, for $C_{f}$/SiBCN CMC, $E_{33}$ and $Z_{T}$ are most affected by changes in porosity, and are similarly reduced to nearly half of their initial value at 20% porosity.
(3) The different locations of the defects are one of the reasons for the dispersion of the mechanical properties of the material at the same porosity. Compared to the longitudinal tensile and longitudinal compressive properties, the variation in boundary porosity significantly affects the transverse tensile, transverse compressive, and shear properties of $C_{f}$/SiBCN CMC, which may be related to the fact that these mechanical properties are more sensitive to the two-phase interface.
(4) Aside from the $E_{33}$, $v_{12}$, $Z_{T}$ and $Z_{C}$ of $C_{f}$/SiBCN CM, the other mechanical properties of the CMCs exhibit a decline as the defect size increases. Without considering the specific shapes of defects, the defects in $C_{f}$/SiBCN CMC should be as small as possible to enhance their mechanical properties.
(5) The mechanical properties of the CMC are more sensitive to the overall porosity, and the mechanical properties can be reduced by half as the overall porosity increases. The mechanical properties of CMC are less sensitive to the size and location of the voids than the overall porosity, and the properties of the material may be reduced by ten percent as the size of the defects increases. To enhance the mechanical properties of $C_{f}$/SiBCN CMC, the primary focus should be on minimizing the total porosity of the composite. Additionally, it is crucial to maximize the matrix infiltration within the fiber tows (yarns). Lastly, ensuring a more uniform matrix filling between the yarns help to reduce defect concentration near the phase interfaces, which can achieve smaller defect sizes under the same porosity.

In conclusion, the research of this paper provides a feasible way to obtain all mechanical properties of CMC for accurate failure analysis of thermal protected structures. Meanwhile, this work identifies the main factors that influence the mechanical properties by defects. First, only spherical defects were considered in this work, and the effect of other shapes of defects on mechanical properties could be further investigated. Second, future experiments under different loading conditions and environments can be carried out to further improve the general applicability of the model. Finally, machine learning methods can also be referenced in future studies to realize rapid prediction of CMC mechanical properties and provide guidance for the design of thermal structures.

## Figures and Tables

**Table 1:** Micro and scale geometry parameters of composites.

Parameter	Value	Parameter	Value
Yarn width	0.68 mm	Fiber volume fraction of fiber tow	65.4%
Yarn spacing	0.76 mm	Fiber volume fraction of composite	40%
Yarn thickness	0.07 mm	Porosity of intra-tow	2.98%
Fabric thickness	0.155 mm	Porosity of inter-tow	20.84%

**Table 3:** Mechanical properties of the constituent materials in 5-harness satin $C_{f}$/SiBCN CMC.

Properties	Fiber Tow	SiBCN Matrix
$E_{11}$ (GPa)	182.2	117
$E_{33}$ (GPa)	34.3	117
$G_{12}$ (GPa)	16.5	48.75
$G_{23}$ (GPa)	7.4	48.75
$v_{12}$	0.213	0.2
$v_{23}$	0.208	0.2
$X_{T}$ (MPa)	1582.8	190
$X_{C}$ (MPa)	2574.1	1900
$Z_{T}$ (MPa)	216.5	190
$Z_{C}$ (MPa)	1500.9	1900
$S_{xy}$ (MPa)	44.2	50
$S_{yz}$ (MPa)	43.9	50

**Table 4:** Predicted and experimental results of mechanical properties of 5-harness satin $C_{f}$/SiBCN CMC.

Properties	Predicted Value	Experiment	Error (%)
$E_{11}$ (GPa)	78.56	82.79 ^*a*^ (2.72 ^*b*^)	5.11
$G_{12}$ (GPa)	15.82	15.36 ^*a*^ (1.71 ^*b*^)	2.99
$X_{T}$ (MPa)	293.1	301.97 ^*a*^ (20.81 ^*b*^)	2.94
$S_{xy}$ (MPa)	52.51	53.65 ^*a*^ (2.27 ^*b*^)	2.12

^*a*^ Mean value; ^*b*^ Standard deviation.

**Table 5:** Parameters of models with different porosity of intra-tow to porosity of inter-tow ratios.

Model	Porosity of Intra-Tow	Porosity of Inter-Tow
A1	11.91	11.91
A2	8.93	14.89
A3	5.96	17.86
A4	2.98	20.84
A5	0	23.82

**Table 6:** Mechanical properties of models with different intra-tow/inter-tow porosity ratios.

Properties	A1	A2	A3	A4	A5
$E_{11}$ (GPa)	79.20	78.82	78.79	78.56	78.25
$E_{33}$ (GPa)	25.58	26.30	24.69	21.53	17.01
$G_{12}$ (GPa)	15.80	15.83	15.66	15.47	15.46
$G_{23}$ (GPa)	10.29	10.45	9.91	8.44	7.01
$v_{12}$	0.106	0.101	0.097	0.091	0.090
$v_{23}$	0.219	0.224	0.229	0.236	0.238
$X_{T}$ (MPa)	270.6	277.6	283.2	293.1	333.1
$X_{C}$ (MPa)	731.4	732.0	765.9	822.2	939.1
$Z_{T}$ (MPa)	103.3	105.9	102.2	92.5	78.7
$Z_{C}$ (MPa)	1556.1	1596.9	1491.1	1313.6	1056.3
$S_{xy}$ (MPa)	48.2	49.9	51.2	52.5	67.2
$S_{yz}$ (MPa)	29.5	32.9	35.2	34.6	32.3

**Table 7:** Parameters of $C_{f}$/SiBCN CMC models with different porosity of boundary.

Model	Porosity of Fiber Tow Boundary	Porosity of the Remaining Matrix
CMC1	0	10
CMC2	2.5	7.5
CMC3	5	5
CMC4	7.5	2.5
CMC5	10	0

**Table 8:** Mechanical properties of $C_{f}$/SiBCN CMC models with different porosity of boundary.

Properties	CMC1	CMC2	CMC3	CMC4	CMC5
$E_{11}$ (GPa)	100.16	100.01	99.93	100.04	100.22
$E_{33}$ (GPa)	40.95	45.27	46.96	45.97	42.19
$G_{12}$ (GPa)	23.53	23.63	23.77	23.93	24.14
$G_{23}$ (GPa)	7.13	7.41	7.65	7.85	8.01
$v_{12}$	0.1100	0.1096	0.1098	0.1103	0.1121
$v_{23}$	0.2189	0.2159	0.2162	0.2182	0.2214
$X_{T}$ (MPa)	412.24	414.92	413.43	413.03	408.99
$X_{C}$ (MPa)	1312.1	1321.0	1340.4	1360.1	1376.9
$Z_{T}$ (MPa)	172.01	191.28	201.39	199.36	188.44
$Z_{C}$ (MPa)	2006.4	2094.6	2111.9	2108.4	2052.0
$S_{xy}$ (MPa)	85.0	85.3	85.8	86.0	86.3
$S_{yz}$ (MPa)	63.7	68.1	70.1	69.6	67.0

**Table 9:** Predicted results of mechanical properties of 5-harness satin $C_{f}$/SiBCN CMC.

Properties	5-Harness Satin $C_{f}$/SiBCN CMC
$E_{11}$ (GPa)	78.56
$E_{33}$ (GPa)	21.53
$G_{12}$ (GPa)	15.82
$G_{23}$ (GPa)	8.44
$v_{12}$	0.091
$v_{23}$	0.236
$X_{T}$ (MPa)	293.1
$X_{C}$ (MPa)	822.2
$Z_{T}$ (MPa)	92.5
$Z_{C}$ (MPa)	1313.6
$S_{xy}$ (MPa)	52.5
$S_{yz}$ (MPa)	34.6

**Table 10:** Predicted and experimental results of failure load of thermal structure.

	Predicted Value	Experiment	Error (%)
Failure load (kN)	19.7	20.4	3.43

## Data Availability

The original contributions presented in this study are included in the article. Further inquiries can be directed to the corresponding author.
